# Dynamic Model and Characteristic Analysis of Viscosity-Ultraelasticity for Bionic Vascular Network

**DOI:** 10.1155/2021/8867150

**Published:** 2021-01-25

**Authors:** Yanli Chen, Xueqing Zhang, Zhiyue Sang, Yongbai Sha, Guiqiang Bai

**Affiliations:** Key Laboratory of CNC Equipment Reliability, Ministry of Education, School of Mechanical and Aerospace Engineering, Jilin University, Changchun 130022, China

## Abstract

Due to the large volume of pipeline transportation, low cost, safety and the reliability, and automatic control, it is widely used in many fields of industrial development and human daily life. Most of the traditional hydraulic pipelines are steel pipes, and their structure is simple. High resistance and high consumption during transportation are not conducive to the sustainable development of society. However, the human vascular system is intricate and has excellent mechanical properties. Built on the review, research on the fluid-solid coupling characteristics of a single bionic pipeline and piping system was carried out. In order to simulate the mechanical characteristics of a fluid conveying pipeline, a fluid-structure coupling model of equation 14 of a single pipeline and the transfer matrix of the pipeline system were established. The mechanical characteristics of the pipeline are studied, and the formula is calculated. The simulation analysis shows that the axial force and flow resistance decrease first and then stabilize with the increase of frequency. Finally, the experimental verification and the results show that the method is both reasonable and effective, because the simulation curve and the experimental curve are consistent in trend.

## 1. Introduction

As a typical biological soft tissue, arterial blood vessels do not exhibit a single linear relationship in mechanical properties. The reasons for this phenomenon are as follows:

*(i) The three-layer structure of the pipe wall* [1]. The content of collagen fibers, elastic fibers, and smooth muscles contained in each layer of the tube wall is different from the spatial arrangement, so that the mechanical properties of the tube wall have compliance, viscoelasticity, superelasticity, etc.

*(ii) It has residual stress.* This makes the vessel wall itself has a special stress-strain relationship.

In summary, the blood vessel can be considered as a special fiber composite reinforced pipeline material [2, 3] has a reasonable basis. The research of the pipeline is derived from the water hammer theory, supplemented by hydrodynamics and dynamics. The equation developed under the classic water hammer theory has the advantages of concise formulas and easy calculation, but fails to take into account the interaction between fluid and pipeline, resulting in a big problem in pipeline dynamic analysis, which is different from the actual pipeline. Largely, it is necessary to establish an accurate pipeline model. Based on the pipeline model developed so far, this paper establishes a bionic pipeline model. Then, solve the pipeline equation and analyze the flow resistance characteristics of the pipeline.

For the pipeline dynamics model, the fluid-structure coupling problem has experienced a mature process from equation (4) to equation (8) and then to equation (14). At present, its solution uses the block Gauss-Seidel implicit scheme and the block method to establish a flow solid coupling solver, using finite volume method to discretize fluid flow problems, and adaptive time step [4, 5], or using fluid-structure interaction (FSI) isogeometric, immersive and fully implicit Calculation method, but this method focuses on viscous incompressible flow and nonlinear superelastic incompressible solids [6]. Pavlou and Dimitrios G. 1 used 14 differential equations to describe the longitudinal-bending-torsion dynamic behavior of liquid-filled pipes, which can be used in the frequency domain or time domain (characteristic method (MOC)) [7]. The four-equation model couples the pipe transient flow with the axial motion of the pipe wall, ignoring the radial inertia, bending, and torsion of the pipe system [8–10], so this paper chooses 14 equations [6, 11–14] to establish a single-root bionic piping; for the solution method of fluid-structure coupling effect, it is necessary to consider factors such as noise and branching [15–17], solve the pipeline equation in the frequency domain, analyze the flow resistance characteristics of the pipeline, and then, study the hydraulic pressure on this basis. For piping system, based on bending moment balance, force balance, and fluid continuity conditions at the branch pipe nodes, a general formula for branch pipe dynamic transfer matrix is established, and the frequency domain transfer matrix method for the dynamic response of liquid-filled manifolds is calculated. The influence of different branch angles and positions, the forced vibration of the branch, is analyzed [18]. The precise integration method is used to solve the transfer matrix, analyze the influence of different piping systems on fluid mechanics characteristics, and establish a bionic piping model to study its pulsation absorption effect [19]. Now, for special-shaped piping such as T-shaped pipe, U-shaped pipe, and curved pipe [20–22], the research is also improving day by day.

This paper uses bionic resources to carry out multiscale, multiparameter, and multi-index similar mechanism and optimization design for the infusion pipeline system and the human blood circulation system, thereby solving the shortcomings of modern hydrostatic transmission systems such as simple structure and single function. Based on the water hammer theory, arterial stress-strain relationship, and fluid mechanics, the 14 equations of a single bionic tube and the transfer matrix of the hydraulic pipeline system are established. This paper uses PDMS and silica gel to simulate the excellent mechanical properties of blood vessels and finally establishes a bionic pipeline experiment to verify the theory of this paper.

## 2. Dynamic Model of a Single Bionic Pipeline

### 2.1. The Establishment of the Bionic Pipeline Model

**Figure 1:**
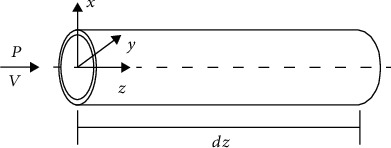
Bionic straight tube microsection force diagram.

**Figure 2:**
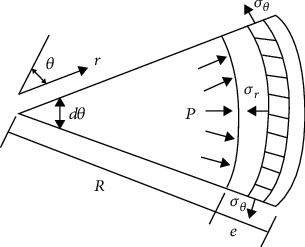
Force diagram of radial section of pipeline.

Blood vessels have very high nonlinear properties. From a mechanical point of view, they are all nonlinear viscoelastic, anisotropic, and heterogeneous materials. This chapter combines the continuum mechanics, uses the strain energy density function, the viscoelastic constitutive model of the component vessel, to simulate the structure and mechanical properties of the blood vessel, obtains the stress-strain relationship of the real vessel wall, and brings it into the pipeline 14 equations [14, 15]; you can get the bionic pipeline dynamics model. Take the microsegment of a single straight bionic pipe for force analysis (as shown in Figure 1). The gravity of the pipe itself and the friction between the fluid and the pipe are ignored during the analysis. Then, according to Figure 2 to analyze the force on the radial section of the pipeline, the axial equations can be generated.

According to the constitutive equation of the blood vessel and the stress-strain relationship, please refer to Appendix A (A.1) and (A.2). The bionic hydraulic pipeline is designed and solved in the frequency domain.

Axial vibration equation can be obtained.

(1) $$\begin{array}{c} \left\{\begin{array}{c} \frac{\partial f_{z}}{\partial z}+\rho_{g}A_{g}\frac{\partial \overset{•}{w_{z}}}{\partial t}=0,\\ \frac{\partial P}{\partial z}+\rho_{l}\frac{\partial V}{\partial t}=0,\\ \frac{\partial \overset{•}{w_{z}}}{\partial z}+\frac{1}{{EA}_{g}}\frac{\partial f_{z}}{\partial t}+\frac{\upsilon }{E}\frac{6\lambda_{\theta }^{2}\lambda_{z}R-3H}{4H}\frac{\partial P}{\partial t}=0,\\ D\frac{\partial P}{\partial t}+\frac{2\upsilon }{{EA}_{g}}\frac{\partial f_{z}}{\partial t}+\frac{\partial V}{\partial z}=0. \end{array}\right. \end{array}$$

Lateral equation can be obtained.

(2) $$\begin{array}{c} \left\{\begin{array}{c} \frac{\partial \overset{•}{w_{x,\text{y}}}}{\partial z}-\overset{•}{\theta_{x,y}}+\frac{1}{{kGA}_{g}}\frac{\partial f_{x,y}}{\partial t}=0,\\ \frac{\partial \overset{•}{\theta_{x,y}}}{\partial z}+\frac{1}{{EI}_{g}}\frac{\partial m_{x,y}}{\partial t}=0,\\ \frac{\partial f_{x,y}}{\partial z}+\left(\rho_{g}A_{g}+\rho_{l}A_{l}\right)\frac{\partial \overset{•}{w_{x,y}}}{\partial t}=0,\\ \frac{\partial m_{y}}{\partial z}+f_{x}+\left(\rho_{g}I_{g}+\rho_{l}I_{l}\right)\frac{\partial \overset{•}{\theta_{y}}}{\partial t}=0,\\ \frac{\partial m_{x}}{\partial z}-f_{y}+\left(\rho_{g}I_{g}+\rho_{l}I_{l}\right)\frac{\partial \overset{•}{\theta_{x}}}{\partial t}=0. \end{array}\right. \end{array}$$

Torsional dynamic equation can be obtained.

(3) $$\begin{array}{c} \left\{\begin{array}{c} \frac{\partial m_{z}}{\partial z}+\rho_{g}J\frac{\partial \overset{•}{\theta_{z}}}{\partial t}=0,\\ \frac{\partial {\overset{•}{\theta }}_{z}}{\partial z}+\frac{1}{GJ}\frac{\partial m_{z}}{\partial t}=0. \end{array}\right. \end{array}$$

### 2.2. Solution of the Bionic Pipeline Model

The bionic pipeline equations obtained in the time domain are difficult to solve, so the Laplace transform is used to transform them into homogeneous high-order equations and Laplace changes, combined with boundary conditions to solve in the frequency domain; the following equation can be obtained.

(4) $$\begin{array}{c} \left[\begin{array}{c} \Phi_{1}^{*}\\ \Phi_{2}^{*}\\ \Phi_{3}^{*} \end{array}\right]=\left[\begin{array}{cccc} T_{1}e^{r_{1}z} & & & \\ & T_{2}e^{r_{2}z} & & \\ & & T_{3}e^{r_{13}z} & \\ & & & T_{4}e^{r_{14}z} \end{array}\right]\xi +\left[\begin{array}{cccc} {TT}_{1} & & & \\ & {TT}_{2} & & \\ & & {TT}_{3} & \\ & & & {TT}_{4} \end{array}\right]\left[\begin{array}{c} \varphi_{1}\left(z,0\right)\\ \varphi_{2}\left(z,0\right)\\ \varphi_{3}\left(z,0\right) \end{array}\right]. \end{array}$$

Simultaneous equation can be obtained.

(5) $$\begin{array}{c} \left\{\begin{array}{c} \varphi_{1}={\left[f_{z}\left(z,0\right)\;\;{\overset{•}{w}}_{z}\left(z,0\right)\;\;P\left(z,0\right)\;\;V\left(z,0\right)\right]}^{T},\\ \varphi_{2}={\left[\begin{array}{cccc} f_{x,y}\left(z,0\right) & {\overset{•}{w}}_{x,y}\left(z,0\right) & m_{y,x}\left(z,0\right) & {\overset{•}{\theta }}_{y,x}\left(z,0\right) \end{array}\right]}^{T},\\ \varphi_{3}={\left[\begin{array}{cc} m_{z}\left(z,0\right) & \overset{•}{\theta }\left(z,0\right) \end{array}\right]}^{T},\\ \Phi_{1}^{*}={\left[\begin{array}{cccc} f_{z}^{*}\left(z,s\right) & {\overset{•}{w}}_{z}^{*}\left(z,s\right) & P^{*}\left(z,s\right) & V^{*}\left(z,s\right) \end{array}\right]}^{T},\\ \Phi_{2}^{*}={\left[\begin{array}{cc} f_{x,y}^{*}\left(z,s\right) & {\overset{•}{w}}_{x,y}^{*}\left(z,s\right) \end{array}\;m_{y,x}^{*}\left(z,s\right){\overset{•}{\theta }}_{y,x}^{*}\left(z,s\right)\right]}^{T},\\ \Phi_{3}^{*}={\left[\begin{array}{cc} {\overset{•}{\theta }}_{z}^{*} & m_{z}^{*} \end{array}\right]}^{T}, \end{array}\right. \end{array}$$

(6) $$\begin{array}{c} \left\{\begin{array}{c} r_{1},r_{3}=-r_{2},-r_{4}=-\sqrt{\frac{\left[-a\pm \sqrt{a^{2}-4b}\right]}{2}},\\ r_{5},r_{7}=-r_{6},-r_{8}=-\sqrt{\frac{\left[-c\pm \sqrt{c^{2}-4d}\right]}{2}},\\ r_{9},r_{11}=-r_{10},-r_{12}=-\sqrt{\frac{\left[-a\pm \sqrt{c^{2}-4d}\right]}{2}},\\ r_{13}=-r_{14}=-\sqrt{-h},\\ D=\frac{1}{K}+\frac{2}{E}\left(\frac{2\lambda_{\theta }^{2}\lambda_{z}R-H}{2H}-\frac{2\lambda_{\theta }^{2}\lambda_{z}R-H}{4H}\upsilon \right),\\ {TT}_{1}=\left[\begin{array}{cccc} 1/s & & & \\ & 1/s & & \\ & & 1/s & \\ & & & 1/s \end{array}\right],\\ {TT}_{2}=\left[\begin{array}{cccc} \frac{Bs}{{kGA}_{g}+{Bs}^{2}} & 0 & 0 & \frac{{BkGA}_{g}}{{kGA}_{g}+{Bs}^{2}}\\ 0 & \frac{1}{s} & 0 & 0\\ 0 & 0 & \frac{1}{s} & 0\\ -\frac{1}{{kGA}_{g}+B} & 0 & 0 & \frac{Bs}{{kGA}_{g}+{Bs}^{2}} \end{array}\right],\\ {TT}_{3}=\left[\begin{array}{cccc} \frac{Bs}{{kGA}_{g}+{Bs}^{2}} & 0 & 0 & -\frac{{BkGA}_{g}}{{kGA}_{g}+{Bs}^{2}}\\ 0 & \frac{1}{s} & 0 & 0\\ 0 & 0 & \frac{1}{s} & 0\\ \frac{1}{{kGA}_{g}+{Bs}^{2}} & 0 & 0 & \frac{Bs}{{kGA}_{g}+{Bs}^{2}} \end{array}\right],\\ {TT}_{4}=\left[\begin{array}{cc} \frac{1}{s} & 0\\ 0 & \frac{1}{s} \end{array}\right],\\ B=\rho_{g}I_{g}+\rho_{l}I_{l},\;s=-2\pi \xi f+i2\pi f\sqrt{1-\xi^{2}}, \end{array}\right. \end{array}$$

where *ξ* is the column vector determined by the boundary conditions. *A*_*g*_ is the cross-sectional area of the pipe wall, *A*_*l*_ is fluid cross-sectional area, *I*_*g*_ is moment of inertia of pipeline section, *I*_*l*_ is moment of inertia, *f* is internal force, *w* is wall displacement, *P* is fluid pressure, *V* is fluid velocity, *k* is the shear distribution coefficient, *G* is shear modulus, and *H*is wall thickness.

### 2.3. Amplitude-Frequency Characteristics of a Single Bionic Pipeline

**Figure 3:**
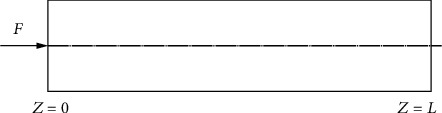
Force diagram of the bionic pipeline

According to the Figure 3, in the case of a plug at the end, *z* = 0 indicates the initial end of the pipeline, and *z* = *L* indicates the end of the pipeline. At the same time, if the pipeline has a plug, the mass of the initial end is *M*_0_, and the mass of the end is *M*_*L*_. Assuming that the two ends of the pipeline are free, there is an ideal axial force of *F* = 9.4KN, and the acting time *T* = 0.002s acts on the end of *z* = 0, and the direction is consistent with the positive direction of the *Z*-axis; thus, the coefficient expression under this boundary condition is obtained.

(7) $$\begin{array}{c} \left[\begin{array}{cccc} O_{1} & O_{2} & O_{3} & O_{4}\\ V_{1} & V_{2} & V_{3} & V_{4}\\ W_{1} & W_{2} & W_{3} & W_{4}\\ Y_{1} & Y_{2} & Y_{3} & Y_{4} \end{array}\right]\left[\begin{array}{c} C_{1}\\ C_{2}\\ C_{3}\\ C_{4} \end{array}\right]+\left[\begin{array}{c} 0\\ -\frac{q_{0}}{s}\\ 0\\ 0 \end{array}\right]=0. \end{array}$$

Simultaneous equation can be obtained.

(8) $$\begin{array}{c} \begin{array}{c} \left\{\begin{array}{c} O_{i}=\frac{-r_{i}}{\rho_{l}s}+\frac{{Er}_{i}}{2v\rho_{g}s^{2}},\\ V_{i}=A_{l}+\frac{{EA}_{g}}{2vs}\left(\frac{r_{i}^{2}}{\rho_{l}s}-Ds\right)-M_{0}s\frac{{Er}_{i}}{2v\rho_{g}s^{2}}\left(\frac{r_{i}^{2}}{\rho_{l}s}-Ds\right),\\ W_{i}=\left[\frac{{Er}_{i}}{2v\rho_{g}s^{2}}\left(\frac{r_{i}^{2}}{\rho_{l}s}-Ds\right)-\frac{r_{i}}{\rho_{l}s}\right]e^{r_{i}z},\\ Y_{i}=\left[A_{l}+\frac{{EA}_{g}}{2vs}\left(\frac{r_{i}^{2}}{\rho_{l}s}-Ds\right)+M_{L}s\frac{{Er}_{i}}{2v\rho_{g}s^{2}}\left(\frac{r_{i}^{2}}{\rho_{l}s}-Ds\right)\right]e^{r_{i}z}, \end{array}\right.\\ i=1,2,3,4. \end{array} \end{array}$$

This time, the program of the bionic pipeline has been improved and a new MATLAB simulation diagram has been obtained. MATLAB simulation was done according to the parameters in Tables 1 and 2 to obtain the amplitude-frequency characteristic curve of the bionic pipeline and steel pipe.

**Figure 4:**
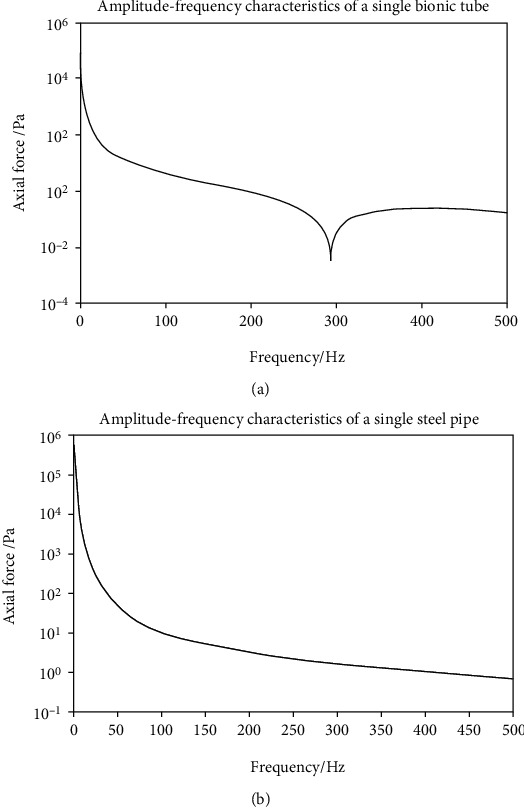
(a) Amplitude-frequency characteristics of bionic pipe. (b) Amplitude-frequency characteristics of stainless steel pipe.

According to Figure 4, no matter how high the frequency is, the axial force-frequency curve of the bionic pipeline is always below the steel pipe, indicating that the bionic pipeline can reduce the energy loss during fluid transportation when fluid is flowing.

According to *P*^∗^(z, s) and *Z*^∗^(z, s) solved by equation (4), combined with specific boundary conditions, the flow resistance characteristics are obtained.

(9) $$\begin{array}{c} Z=\frac{P}{Q}=\frac{C_{1}e^{r_{1}z}+C_{2}e^{r_{2}z}+C_{3}e^{r_{3}z}+C_{4}e^{r_{4}z}}{\left(C_{5}e^{r_{1}z}+C_{6}e^{r_{2}z}+C_{7}e^{r_{3}z}+C_{8}e^{r_{4}z}\right)\pi R^{2}}. \end{array}$$

Simultaneous equation can be obtained.

(10) $$\begin{array}{c} \left\{\begin{array}{c} C_{5}=-\frac{r_{1}}{\rho_{l}s}C_{1},\\ C_{6}=-\frac{r_{2}}{\rho_{l}s}C_{2},\\ C_{7}=-\frac{r_{3}}{\rho_{l}s}C_{3},\\ C_{8}=-\frac{r_{4}}{\rho_{l}s}C_{4}. \end{array}\right.\; \end{array}$$

**Figure 5:**
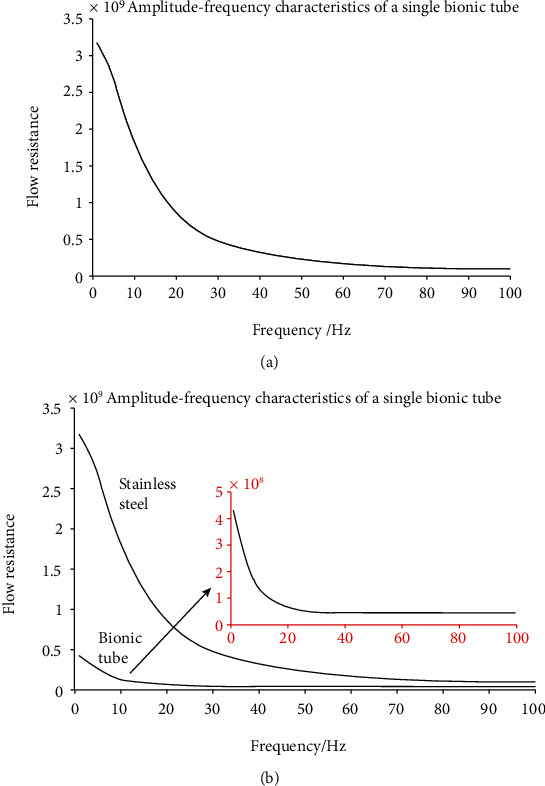
(a) Research on flow resistance characteristics of single steel pipe. (b) Research on flow resistance characteristics of single steel pipe and bionic pipe.

According to Figure 5, the flow resistance characteristic curve of the steel pipe is on the top of the bionic tube. The bionic tube is made of PDMS. PDMS has good rheology, and the elastic modulus and Poisson's ratio are similar to those of human blood vessels. The mechanical properties can achieve blood vessel viscosity reduction, the effect of resistance.

## 3. Bionic Pipeline System Solution Analysis

This section takes the straight pipe system, the variable diameter pipe system, and the diversion pipe system as examples to study the state variables at any position, which is of great significance to the pipeline flow.

### 3.1. Bionic Straight Pipe System

There are several commonly used methods for solving the transfer matrix of straight pipelines: characteristic line method [23], finite element method [24], immersive interpolation method, and transfer matrix method [25]. The precise integration method has high-accuracy, high-efficiency, unlimited solution in the fluid-structure coupling solution algorithm and can obtain a high-precision transfer matrix. Therefore, the precise integration method is used to solve the field transfer matrix. The core of solving the transfer matrix of a straight pipe system is to use Taylor series expansion and Pader series expansion [26] to solve. The Pader approximation is often more accurate than the truncated Taylor series, and when the Taylor series does not converge, Pader approximation is often still feasible, so this paper chooses Pader series to solve.

The transfer matrix equation of the straight tube field of a single bionic pipeline without external excitation:

(11) $$\begin{array}{c} T\left(z,s\right)=e^{\left(\alpha s+\beta \right)z}. \end{array}$$

Let *U* = *αs* + *β*, then the Pader approximation of *T*(z, s) = *e*^*Uz*^ is

(12) $$\begin{array}{c} T_{q1}\left({U_{1}}^{\prime }\right)=I+R_{a}. \end{array}$$

Simultaneous equation can be obtained.

(13) $$\begin{array}{c} \left\{\begin{array}{c} R_{a}={\left(I+D_{a}\right)}^{-1}\left(N_{a}-D_{a}\right),\\ {U_{1}}^{\prime }=\frac{{\text{U}}_{1}}{2^{N}},\\ N_{a}=\overset{q}{\underset{k=1}{\sum }}\frac{\left(2q-k\right)!q!}{\left(2q\right)!k!\left(q-k\right)!}{\text{U}}_{1}\prime^{k},\\ D_{a}=\overset{q}{\underset{k=1}{\sum }}\frac{\left(2q-k\right)!q!}{\left(2q\right)!k!\left(q-k\right)!}{\left(-{U_{1}}^{\prime }\right)}^{k}. \end{array}\right. \end{array}$$

The choice of parameters *N* and *q* directly affects the accuracy of the exponential matrix. If the initial value of *q* = *q*_*s*_ = 1, *N* = *N*_*s*_ is set, the error analysis requirement is met.

(14) $$\begin{array}{c} N_{0}=\max\left(0,\log_{2}^{{\left\|U_{1}\right\|}_{\infty }}+1\right). \end{array}$$

When a single bionic pipeline is externally excited, its field transfer matrix is

(15) $$\begin{array}{c} \Omega \left(0,s\right)=T\left(z,s\right)\Omega \left(z,s\right)+ϒ\left(z,s\right). \end{array}$$

Among them, *ϒ*(*z*, *s*) represents the state matrix of external excitation.

Assuming that the straight pipe piping system is an integral pipe with a total length of *L* composed of the same single bionic in section *n*, the initial and ending state vectors of the pipeline are *Π*_*s*_ and *Π*_*e*_, and the *i* section is *Π*_*s*_^*i*^ and *Π*_*e*_^*i*^. The total transfer matrix is

(16) $$\begin{array}{c} \Omega_{s}=T_{1}\left(z_{1},s\right)\Omega_{e1}+{ϒ}_{1}\left(z_{1},s\right)=T_{1}\left(T_{2}\left(z_{2},s\right)\Omega_{e2}+{ϒ}_{2}\left(z_{2},s\right)\right)+{ϒ}_{1}\left(z_{1},s\right)=\cdots =T_{1}T_{2}\cdots T_{n-1}T_{n}\Omega_{e}+T_{1}T_{2}\cdots T_{n-1}{ϒ}_{3}\left(z_{n},s\right)+\cdots T_{1}{ϒ}_{2}\left(z_{2},s\right)+{ϒ}_{1}\left(z_{1},s\right)=\text{T}\left(n\right)\Omega_{e}+ϒ\left(n-1\right). \end{array}$$

Simultaneous equation can be obtained.

(17) $$\begin{array}{c} \left\{\begin{array}{c} \text{T}\left(n\right)=T_{1}T_{2}\cdots T_{n}=\overset{n}{\underset{i=1}{\prod }}T_{i},\\ ϒ\left(n-1\right)=\overset{n-1}{\underset{i=0}{\sum }}\left[\text{T}\left(i\right){ϒ}_{i}\left(z_{i},s\right)\right], \end{array}\right. \end{array}$$

where *z* is the length of the pipeline and the subscript *W* represents the count of the pipeline. Combining the transfer relationship between boundary conditions and pipelines, the state vector *Ω*(*z*, *s*) at any point of the bionic straight pipeline system can be obtained.

### 3.2. Fractal Piping System

In the process of flow transportation, variable-diameter pipelines are also widely used, mostly in oil and gas gathering and transportation pipeline networks in oil and gas fields and urban water supply or gas distribution pipeline networks. The reliability of transportation is higher than that of the branched pipe network, and the transportation after that point will not be interrupted due to a failure.

**Figure 6:**
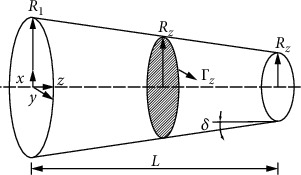
Conical pipeline model.

Assuming *R*_2_ < *R*_1_, the wall thickness is *h*. From Figure 6, the transfer matrix equation can be obtained.

(18) $$\begin{array}{c} \left\{\begin{array}{c} \frac{\partial T^{-1}\left(z,s\right)}{\partial z}+U\left(z\right)T^{-1}\left(z,s\right)=0,\\ T\left(0,s\right)=I,U\left(z\right)=\left(\alpha \left(z\right)s+\beta \left(z\right)\right). \end{array}\right. \end{array}$$

Using the idea of finite element, the pipeline of length *z* is divided into *n* sections, and each section is Δ*z* = *z*/*n*, that is, when Δ*z*⟶0, the coefficient matrix is approximately equivalent to a constant, and the exponential matrix *U*_*i*_(*i* · Δ*z*) can also be approximately regarded as a constant matrix. So, the overall transfer matrix of the pipeline system is as follows:

(19) $$\begin{array}{c} T=\overset{n}{\underset{i=1}{\prod }}T_{i}=\overset{n}{\underset{i=1}{\prod }}e^{\left[U\left(i-\left(1/2\right)\Delta z\right)\Delta z\right]}=e^{\left[\Delta z\overset{n}{\underset{i=1}{\sum }}U\left(i-\left(1/2\right)\Delta z\right)\Delta z\right]}, \end{array}$$

(20) $$\begin{array}{c} T_{q2}\left({U_{2}}^{\prime }\right)=I+R_{a}. \end{array}$$

Simultaneous equation can be obtained.

(21) $$\begin{array}{c} \left\{\begin{array}{c} R_{a}={\left(I+D_{a}\right)}^{-1}\left(N_{a}-D_{a}\right),\\ {U_{2}}^{\prime }=\frac{{\text{U}}_{2}}{2^{N}}=\frac{\sum_{i=1}^{n}U\left(i-\left(1/2\right)\Delta z\right)\Delta z}{2^{N}},\\ N_{a}=\overset{q}{\underset{k=1}{\sum }}\frac{\left(2q-k\right)!q!}{\left(2q\right)!k!\left(q-k\right)!}{\text{U}}_{2}\prime^{k},\\ D_{a}=\overset{q}{\underset{k=1}{\sum }}\frac{\left(2q-k\right)!q!}{\left(2q\right)!k!\left(q-k\right)!}{\left(-{U_{2}}^{\prime }\right)}^{k}. \end{array}\right. \end{array}$$

Similar to the straight pipe system, setting the initial values *q* = *q*_*s*_ = 1 and *N* = *N*_*s*_ to meet the error analysis requirements is

(22) $$\begin{array}{c} N_{0}=\max\left(0,\log_{2}^{{\left\|U_{2}\right\|}_{\infty }}+1\right). \end{array}$$

### 3.3. Diversion Piping System

**Figure 7:**
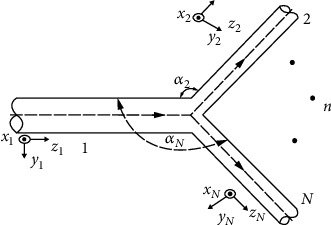
Multisection branch pipeline model.

Branch pipeline is a more complicated kind of pipeline, which plays a role of splitting or merging. According to the Figure 7, common forms include T-shaped pipeline and Y-shaped pipeline. Solving the transfer matrix of the shunt pipeline needs to adopt the absorption transfer matrix method, which is different from the straight pipe pipeline and the variable diameter pipeline system. According to the balance of each branch force and moment and the continuity of the fluid, a set of equations is established, and then, Laplace transformation is performed to obtain the transfer matrix of the branch pipeline system.

Transfer matrix between pipeline 1 and pipeline 2~*N*:

(23) $$\begin{array}{c} Q_{1}\Omega_{1}=Q_{1}\Omega_{1}+Q_{2}\Omega_{2}+\cdots Q_{N}\Omega_{N}. \end{array}$$

In the formula, *Ω*_1_, *Ω*_2_, *Ω*_3_ ⋯ *Ω*_*N*_ (3 ≤ *n* ≤ *N*) represents the state vector of each pipeline branch point, it is the coefficient matrix of 14 × 14, and the matrix form is shown in (A.3) and (A.4) of Appendix A.

(24) $$\begin{array}{c} Q_{1}=\left[\begin{array}{cc} \begin{array}{cccc} 1 & A_{l} & 0 & 0\\ 0 & 1 & 0 & 0\\ 0 & 0 & 1 & 0\\ 0 & 0 & -A_{l} & A_{l} \end{array} & O\\ O & E \end{array}\right]. \end{array}$$

Transfer matrix between pipeline 2 and pipeline *N*:

(25) $$\begin{array}{c} Q_{n}^{\prime }\Omega_{n}=Q_{2}^{\prime }\Omega_{2}\;\left(2\leq n\leq N\right). \end{array}$$

In the formula, *Ω*_1_, *Ω*_2_, *Ω*_3_ ⋯ *Ω*_*N*_ (3 ≤ *n* ≤ *N*) represents the state vector of each pipeline branch point. *Q*_2_′, *Q*_3_′ ⋯ *Q*_*N*_′(2 ≤ *n* ≤ *N*) is a 14 × 14 coefficient matrix; the matrix form is shown in (A.5) of Appendix A. It can be known from the absorption transfer matrix that, assuming that pipelines 1-2 is the main transfer path, referring to the straight pipeline transfer matrix, the following equation can be obtained.

(26) $$\begin{array}{c} \left\{\begin{array}{c} I_{n}=\left(\begin{array}{c} Q_{n}^{\prime }\\ H_{n}T_{n}^{-1} \end{array}\right),\\ R=\left(\begin{array}{c} Q_{2}^{\prime }\\ 0 \end{array}\right),\\ {ℵ}_{n}=\left(\begin{array}{c} 0\\ F_{n-e}+H_{n}T_{n}^{-1}{ϒ}_{n} \end{array}\right). \end{array}\right. \end{array}$$

Get the shunt transfer matrix.

(27) $$\begin{array}{c} \Omega_{n}=I_{n}^{-1}R\Omega_{2}+I_{n}^{-1}{ℵ}_{n}, \end{array}$$

(28) $$\begin{array}{c} \Omega_{1}=Q_{1}^{-1}\left[Q_{2}+Q_{3}I_{3}^{-1}R+\cdots +Q_{N}I_{3}^{-1}R\right]\Omega_{2}+Q_{1}^{-1}\left[Q_{3}I_{3}^{-1}{ℵ}_{3}+\cdots +Q_{N}I_{N}^{-1}{ℵ}_{N}\right]. \end{array}$$

In the formula, *T*_*Q*_ = *Q*_1_^−1^[*Q*_2_ + *Q*_3_*ℑ*_3_^−1^*ℜ*+⋯+*Q*_*N*_*ℑ*_3_^−1^*ℜ*] is the point transfer matrix, and *Q*_*n*_*ℑ*_*n*_^−1^ represents the main path is affected by the *n*th pipeline. This method of solving the transfer matrix is called the “absorption transfer matrix method” [16].

## 4. Experimental Design of Bionic Pipeline

**Figure 8:**
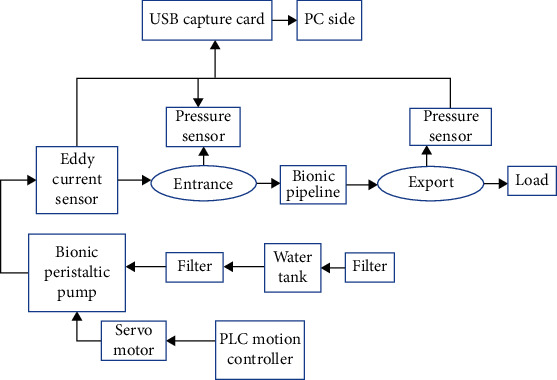
Experimental design.

The schematic diagram of this experiment is shown in Figure 8 [27, 28], and the experimental equipment parameters are shown in Table 3. The comparative experiment shown in Table 4 was established. Polydimethylsiloxane (PDMS, polydimethylsiloxane) has good light permeability and high structural elasticity and is relatively better prepared. And the elastic modulus and Poisson's ratio [29] of the material PDMS are similar to the data derived from the previous article, so it is selected as the best test material. The outer diameter of the design test bionic pipeline model is 14 mm, the wall thickness is 2 mm, and the length is 300 mm.

**Figure 9:**
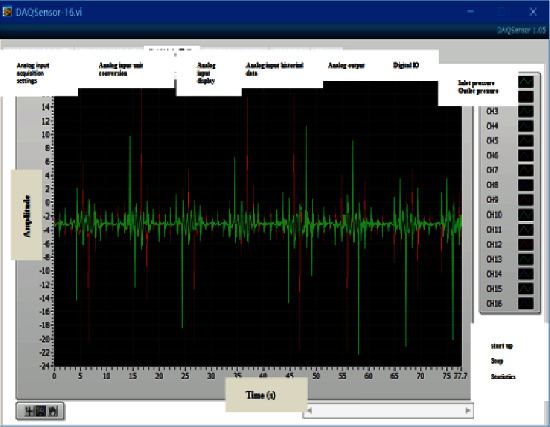
Real-time interface of acquisition status.

The DAQ sensor data acquisition card with a maximum sampling frequency of 250 kHz is selected. The real-time interface of the acquisition status is shown in Figure 9. Need to mark the meaning of the channel, and set the save path. This test only needs to check 8 channels.

**Figure 10:**
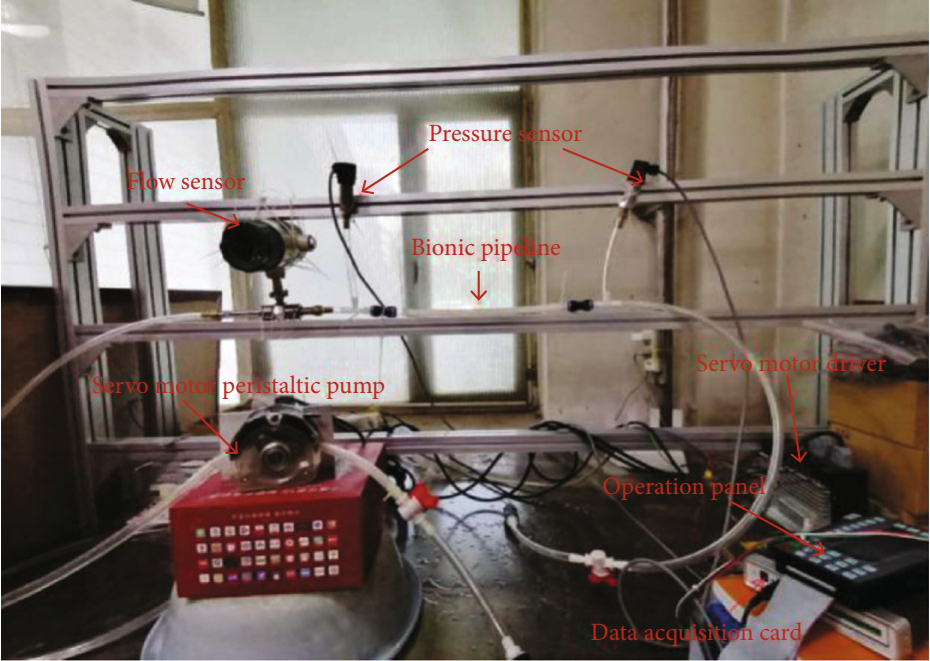
Experimental table.

**Figure 11:**
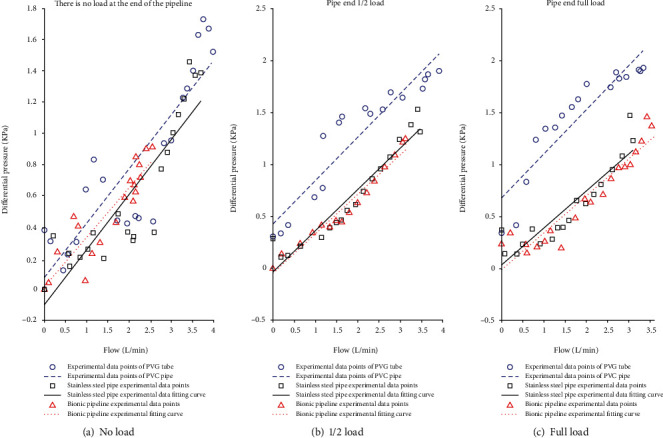
Flowing liquid-purified water.

**Figure 12:**
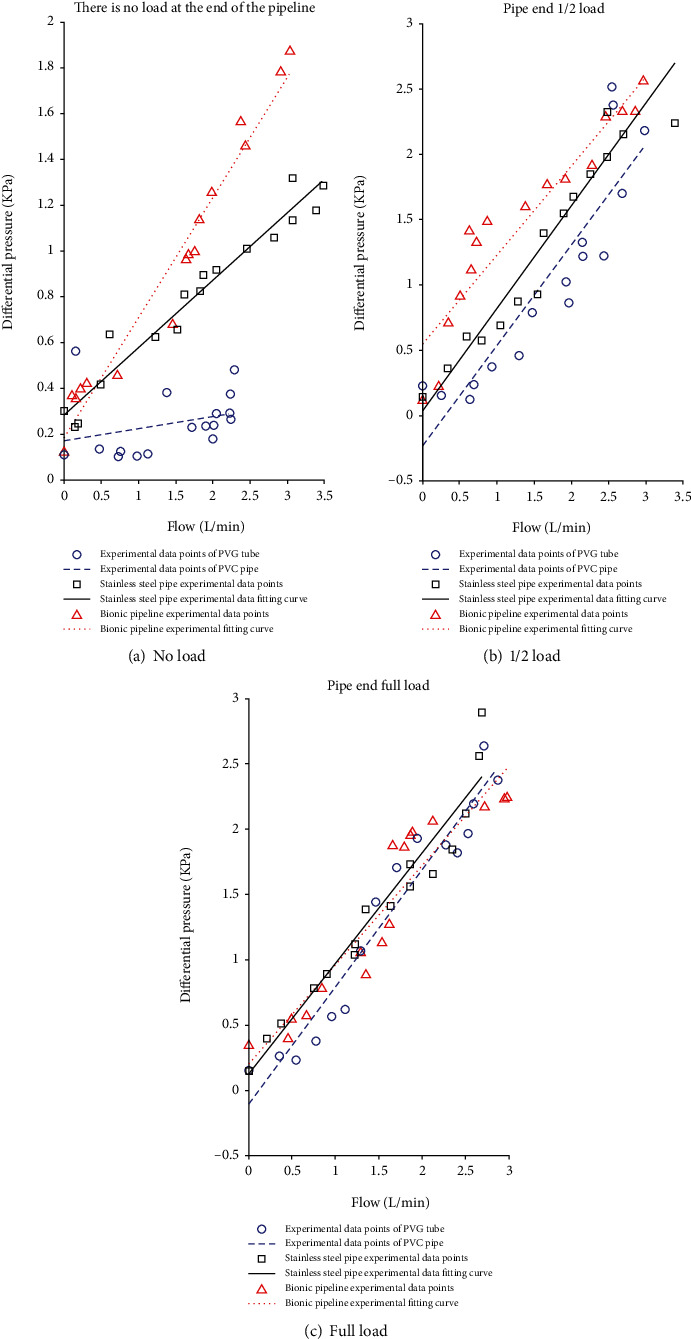
Infusion liquid-glycerol solution.

**Figure 13:**
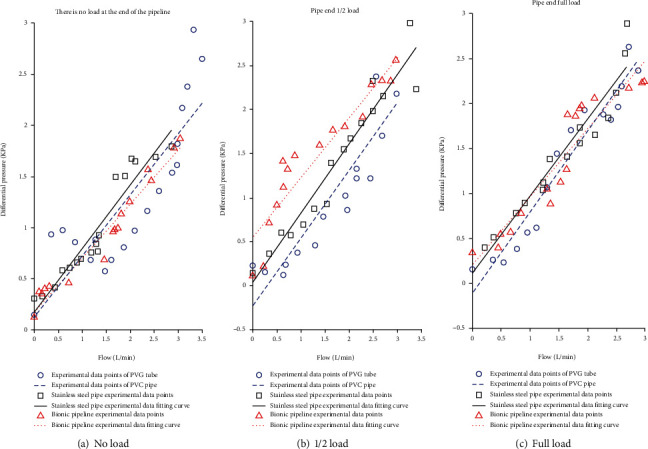
Inflow liquid-hydraulic oil.

The built test bed is illustrated in Figure 10. In the preparation of the bionic tube, the PDMS solution is mixed in proportion, poured into a vacuum pump to evacuate, and the mold release agent is evenly sprayed in the mold, and then, the pumped solution is poured into the mold In the process, it is fixed and placed vertically with a fixture and finally cooled to room temperature. After about 8 hours, the liquid is solidified and molded, and it can be carefully demolded and taken with the release agent. Adjust the throttle valve opening to the fully open state; at this time, it means that the pipeline only carries flow and does not bear pressure. Because air bubbles will cause test errors, turn on the CNC SN4100K and adjust it to a higher speed to quickly exhaust the air in the system. After the air in the pipeline is exhausted, manually suspend the CNC SN4100K and switch to low-speed mode, and then, you need to be ready to collect data. By adjusting the peristaltic pump, the motor speed is increased steadily in an arithmetic sequence with a tolerance of 30 r/min. With the increase of the speed of the CNC SN4100K, when the rated flow of the peristaltic pump is reached, the flow will stop recording. Change the external load, that is, the throttle valve port size (external load 2 and a half full open, external load 3 microport) and repeat the experiment to record the data. The measured experimental data are simulated with MATLAB, and the experimental results are shown in Figures 11, 12, and 13.

According to equation (9), the fluid resistance in the pipeline is the ratio of the pressure difference *P* to the flow rate *Q*, and the slope of the fitting curve in Figures 11–13 is the fluid resistance. Except for Figure 12(a) (the fluid is a glycerin solution, no load), the flow resistance of the bionic pipeline is greater than the flow resistance in the case of PVC pipes and steel pipes; the rest are the bionic pipelines with the smallest flow resistance, because the length of the comparison pipeline in the experiment only 300 mm plus the system error and the error of the experimental instrument. When the medium in the bionic pipeline is hydraulic oil and glycerin aqueous solution, the slopes of the three pipelines are not much different.

The test results show that the bionic pipeline has advantages in the flow system under the test conditions mentioned above. Taking the case where the flow medium of the bionic pipeline is water as an example, the equation of the fitted curve is

(29) $$\begin{array}{c} y=0.31826x+0.012253. \end{array}$$

**Figure 14:**
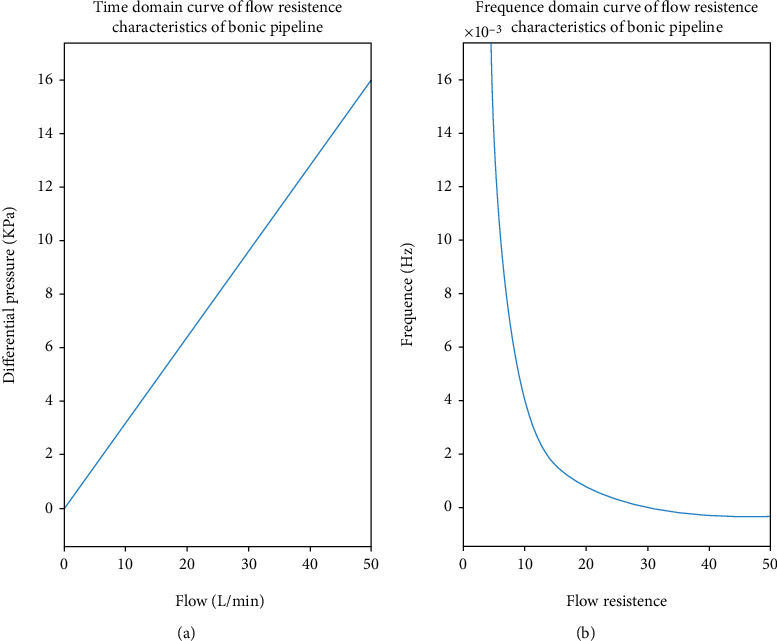
Flow resistance characteristic curve of bionic pipeline. (a) Bionic pipeline flow resistance characteristic curve in time domain. (b) Flow resistance characteristic curve of bionic pipeline in frequency domain.

The physical meaning represented by the slope of the equation is the flow resistance. Laplace transform of the curve equation can obtain the flow resistance characteristic curve in the frequency domain of a single bionic pipeline (as shown in Figure 14).

Figure 14(a) shows that when the flow resistance of the fluid in the pipeline is a fixed value, the flow rate of the fluid is proportional to the pressure difference, which is consistent with equation (9), indicating the rationality and correctness of this experiment.  Figure 14(b) is a graph of Figure 14(a) in the frequency domain. As the frequency increases, the flow rate decreases and eventually tends to zero. The results are consistent with the theoretical calculation of the flow resistance characteristics of the bionic pipeline model established in Chapter 2. The reason for the deviation is that the test conditions are limited and the plugs cannot be placed at both ends, and because the bionic pipeline is not easy to prepare, there is the phenomenon that the demoulding is easily damaged makes the pressure drop data not significantly changed within the limited preparation length, which is inconsistent with the theoretical calculation condition length.

## 5. Conclusions

The contributions of this paper are summarized as follows:

1. Based on the theory of continuum mechanics, the blood vessel is considered as a unique matrix-based composite reinforcement material, and the stress-strain relationship of the blood vessel is obtained. On this basis, a single straight bionic pipeline model is established. The use of Laplace change and the conversion of 14 equations of bionic piping into 14 homogeneous high-order frequency domain partial differential equations are introduced in detail. Under certain boundary conditions, the amplitude-frequency response characteristics and flow characteristics of bionic pipelines and steel pipes are analyzed, resistance characteristics
2. To further analyze the complex hydraulic pipeline system, an accurate adaptive parameter selection method is proposed to solve the exponential matrix of pipeline system, in which the linear pipeline and variable diameter pipeline system are used as the research objects based on a single bionic tube. For the more complicated shunt pipeline system, the state vector is solved by the idea of absorption transfer matrix. Set up a peristaltic pump-biomimetic pipeline test bench, establish an orthogonal test, and verify the superiority of the bionic pipeline in the process of flow

## Figures and Tables

**Table 1:** Parameter table.

Parameter category	Table
Sectional area of pipe wall sectional area of pipe wall *A*_*g*_/mm^2^	6.9336 × 10^−4^
Moment of inertia of pipe section *I*_*g*_/mm^2^	2.7261 × 10^−7^
Shear modulus *G*	1.1538 × 10^6^
Shear distribution coefficient *k*	10/9
Fluid cross-sectional area *A*_*l*_/mm^2^	0.0021
Fluid moment of inertia *I*_*l*_/mm^2^	3.5891 × 10^−7^

**Table 2:** Bionic pipeline and fluid physical parameters.

Pipeline		Plug	
Tube length/mm	4.51	Plug quality *M*_0_ (*z* = 0)/kg	1.312
Inner diameter R/mm	26	Plug quality *M*_*L*_(*z* = l)/kg	0.3258
Wall thickness H/mm	3.95	Fluid	
Young's modulus *E*/MPa	1~10	Bulk modulus K/GPa	2.14
Poisson's ratio *ν*	0.49	Fluid density *ρ*_*l*_/(g/cm^3^)	0.999
Pipeline density *ρ*_*g*_/(g/cm^3^)	1.030	Damping ratio *ξ*	0.002

**Table 3:** Test equipment parameter.

Instrument	Parameter				
	Model	Measuring range	Voltage information	Current information	Characteristic
Servo motor	CNCSN4100K		24 V DC power supply		16 input ports, 8 output ports System frequency 100 kHz
Driver	CNC 60 servo motor		200 AC power supply		Rated speed 3000 rpm
Turbine flowmeter	Famit DN6	0.1~0.6 m^3^/h	3.6 V lithium battery power supply	4~20 mA	1.5 grade accuracy
Data acquisition card	USB2610	Maximum working pressure 6.3 MPa		4~20 mA	
Pressure sensor	MIK-P300	0~100 KPa		4~20 mA	Sampling frequency 250 kHz

**Table 4:** Comparison experiment.

Solution	Curve fitting	Pipe		
		Stainless steel pipe	PVC pipe	Bionic pipeline
Pure water	No load	*y*1 = 0.34836 *x* + 0.072843	*y*2 = 0.35427 *x* − 0.10162	*y*3 = 0.31826 *x* + 0.012253
	1/2 load	*y*1 = 0.41896 *x* + 0.4245	*y*2 = 0.39785 *x* − 0.036787	*y*3 = 0.37963 *x* − 0.046299
	Full load	*y*1 = 0.42499 *x* + 0.67528	*y*2 = 0.35707 *x* + 0.031116	*y*3 = 0.35666 *x* − 0.014652

Glycerin solution	No load	*y*1 = 0.05228 *x* + 0.17294	*y*2 = 0.29299 *x* + 0.28299	*y*3 = 0.52445 *x* + 0.18396
	1/2 load	*y*1 = 0.77126 *x* − 0.23482	*y*2 = 0.78824 *x* + 0.031552	*y*3 = 0.68238 *x* + 0.54698
	Full load	*y*1 = 0.89617 *x* − 0.10496	*y*2 = 0.8466 *x* + 0.12447	*y*3 = 0.75899 *x* + 0.20071

Hydraulic oil	No load	*y*1 = 0.59618 *x* + 0.12593	*y*2 = 0.62191 *x* + 0.16613	*y*3 = 0.52445 *x* + 0.18396
	1/2 load	*y*1 = 0.77126 *x* − 0.23482	*y*2 = 0.78824 *x* + 0.031552	*y*3 = 0.68238 *x* + 0.54698
	Full load	*y*1 = 0.89617 *x* − 0.10496	*y*2 = 0.8466 *x* + 0.12447	*y*3 = 0.75899 *x* + 0.20071

## Data Availability

The data used to support the findings of this study are included within the article. All the data in the supplementary materials have appeared in Figure 11, Figure 12, and Figure 13. The data used in the manuscript is provided as supplementary materials and is superfluous and useless.

## Appendix

### Vascular constitutive model:

$W\left(C,\dot{C},A_{0}\right)=W^{e}\left(C,A_{0}\right)+\psi^{\nu }\left(C,\dot{C},A_{0}\right)$

(A.1) $$\begin{array}{c} W^{e}=W_{m}^{e}+W_{f}^{e}+W_{\kappa }^{e}=\left\{\frac{c_{m}}{2}\left(\bar{I_{1}}-3\right)+\frac{1}{D}\left(\frac{J^{2}-1}{2}-\ln J\right)\right\}+\left\{\frac{k_{1}}{2k_{2}}\left\{e^{k_{2}{\left[\kappa \bar{I_{1}}+\left(1-3\kappa \right)\bar{I_{4}}-1\right]}^{2}}-1\right\}+\frac{k_{1}}{2k_{2}}\left\{e^{k_{2}{\left[\kappa \bar{I_{1}}+\left(1-3\kappa \right)\bar{I_{6}}-1\right]}^{2}}-1\right\}\right\}, \end{array}$$

(A.2) $$\begin{array}{c} \frac{\sigma_{z}}{\lambda_{z}}=\frac{c_{m}}{2}\left[\lambda_{\theta }^{2}+2\lambda_{z}{\dot{\lambda }}_{z}\right]-2\lambda_{\theta }^{-2}\lambda_{z}^{-3}{\dot{\lambda }}_{z}+\left\{\begin{array}{cc} 0, & I_{4}\leq 1,\\ 4c_{2}\lambda_{z}{\dot{\lambda }}_{z}\sin^{2}\gamma \left[\lambda_{z}^{2}\sin^{2}\gamma +\lambda_{\theta }^{2}\cos^{2}\gamma -1\right]+ & \\ 8c_{2}\lambda_{z}{\dot{\lambda }}_{z}\sin^{2}\gamma {\left[\lambda_{z}^{2}\sin^{2}\gamma +\lambda_{\theta }^{2}\cos^{2}\gamma -1\right]}^{3}- & \\ 4k_{1}e^{k_{2}{\left[\kappa I_{1}+\left(1-3\kappa \right)I_{4}-1\right]}^{2}}\left[\kappa I_{1}+\left(1-3\kappa \right)I_{4}-1\right]\times & \\ \left(\kappa +\sin^{2}\gamma -3\kappa \sin^{2}\gamma \right)\lambda_{z}{\dot{\lambda }}_{z}, & I_{4}>1, \end{array}\right.+\left\{\begin{array}{cc} 2\eta_{1}\left[\left(-2\lambda_{\theta }^{-1}{\dot{\lambda }}_{\theta }-2\lambda_{\theta }^{-5}{\dot{\lambda }}_{\theta }+3\lambda_{\theta }^{-3}{\dot{\lambda }}_{\theta }\right)\lambda_{z}{\dot{\lambda }}_{z}\right], & I_{4}\leq 1,\\ 2\eta_{1}\left[\left(-2\lambda_{\theta }^{-1}{\dot{\lambda }}_{\theta }-2\lambda_{\theta }^{-5}{\dot{\lambda }}_{\theta }+3\lambda_{\theta }^{-3}{\dot{\lambda }}_{\theta }\right)\lambda_{z}{\dot{\lambda }}_{z}\right]+ & \\ 4\eta_{2}{\left(\lambda_{z}{\dot{\lambda }}_{z}\right)}^{3}\left(\lambda_{z}^{2}\sin^{2}\gamma +\lambda_{\theta }^{2}\cos^{2}\gamma -1\right), & I_{4}>1, \end{array}\right. \end{array}$$

(A.3) $$\begin{array}{c} Q_{2}=\left[\begin{array}{cccccccccccccc} -\cos\alpha_{2} & -A_{l2}\cos\alpha_{2} & 0 & 0 & 0 & 0 & 0 & 0 & 0 & 0 & \sin\alpha_{2} & 0 & 0 & 0\\ 0 & 1 & 0 & 0 & 0 & 0 & 0 & 0 & 0 & 0 & 0 & 0 & 0 & 0\\ -\cos\alpha_{2} & 0 & 0 & 0 & 0 & 0 & 0 & 0 & \sin\alpha_{2} & 0 & 0 & 0 & 0 & 0\\ 0 & 0 & -A_{l2} & A_{l2} & 0 & 0 & 0 & 0 & 0 & 0 & 0 & 0 & 0 & 0\\ 0 & 0 & -\sin\alpha_{2} & 0 & -\cos\alpha_{2} & 0 & 0 & 0 & 0 & 0 & 0 & 0 & 0 & 0\\ 0 & -A_{l2}\sin\alpha_{2} & 0 & -\sin\alpha_{2} & 0 & -\cos\alpha_{2} & 0 & 0 & 0 & 0 & 0 & 0 & 0 & 0\\ 0 & 0 & 0 & 0 & 0 & 0 & 1 & 0 & 0 & 0 & 0 & 0 & 0 & 0\\ 0 & 0 & 0 & 0 & 0 & 0 & 0 & 1 & 0 & 0 & 0 & 0 & 0 & 0\\ 0 & 0 & 0 & 0 & 0 & 0 & 0 & 0 & 1 & 0 & 0 & 0 & 0 & 0\\ 0 & 0 & 0 & 0 & 0 & 0 & 0 & 0 & 0 & 1 & 0 & 0 & 0 & 0\\ 0 & 0 & 0 & 0 & 0 & 0 & 0 & 0 & 0 & 0 & -\cos\alpha_{2} & 0 & -\sin\alpha_{2} & 0\\ 0 & 0 & 0 & 0 & 0 & 0 & 0 & 0 & 0 & 0 & 0 & -\cos\alpha_{2} & 0 & -\sin\alpha_{2}\\ 0 & 0 & 0 & 0 & 0 & 0 & 0 & 0 & 0 & 0 & \sin\alpha_{2} & 0 & -\cos\alpha_{2} & 0\\ 0 & 0 & 0 & 0 & 0 & 0 & 0 & 0 & 0 & 0 & 0 & \sin\alpha_{2} & 0 & -\cos\alpha_{2} \end{array}\right], \end{array}$$

(A.4) $$\begin{array}{c} Q_{N}=\left[\begin{array}{cccccccccccccc} -\cos\alpha_{n} & -A_{l}\cos\alpha_{n} & 0 & 0 & 0 & 0 & 0 & 0 & 0 & 0 & \sin\alpha_{n} & 0 & 0 & 0\\ 0 & 0 & 0 & 0 & 0 & 0 & 0 & 0 & 0 & 0 & 0 & 0 & 0 & 0\\ 0 & 0 & 0 & 0 & 0 & 0 & 0 & 0 & 0 & 0 & 0 & 0 & 0 & 0\\ 0 & 0 & -A_{l} & A_{l} & 0 & 0 & 0 & 0 & 0 & 0 & 0 & 0 & 0 & 0\\ 0 & 0 & 0 & 0 & 0 & 0 & 0 & 0 & 0 & 0 & 0 & 0 & 0 & 0\\ 0 & 0 & 0 & 0 & 0 & 1 & 0 & 0 & 0 & 0 & 0 & 0 & 0 & 0\\ 0 & 0 & 0 & 0 & 0 & 0 & 1 & 0 & 0 & 0 & 0 & 0 & 0 & 0\\ 0 & 0 & 0 & 0 & 0 & 0 & 0 & -\cos\alpha_{n} & 0 & 0 & 0 & 0 & -\sin\alpha_{n} & 0\\ 0 & 0 & 0 & 0 & 0 & 0 & 0 & 0 & 0 & 0 & 0 & 0 & 0 & 0\\ 0 & 0 & 0 & 0 & 0 & 0 & 0 & 0 & 0 & 0 & 0 & 0 & 0 & 0\\ -\sin\alpha_{n} & -A_{l}\sin\alpha_{n} & 0 & 0 & 0 & 0 & 0 & 0 & 0 & 0 & -\cos\alpha_{n} & 0 & 0 & 0\\ 0 & 0 & 0 & 0 & 0 & 0 & 0 & 0 & 0 & 0 & 0 & 1 & 0 & 0\\ 0 & 0 & 0 & 0 & 0 & 0 & 0 & \sin\alpha_{n} & 0 & 0 & 0 & 0 & 0 & -\cos\alpha_{n}\\ 0 & 0 & 0 & 0 & 0 & 0 & 0 & 0 & 0 & 0 & 0 & 0 & 0 & 0 \end{array}\right], \end{array}$$

(A.5) $$\begin{array}{c} Q_{n}^{\prime }=\left[\begin{array}{cccccccccccccc} 0 & 1 & 0 & 0 & 0 & 0 & 0 & 0 & 0 & 0 & 0 & 0 & 0 & 0\\ 0 & 0 & \cos\alpha_{n} & 0 & -\sin\alpha_{n} & 0 & 0 & 0 & 0 & 0 & 0 & 0 & 0 & 0\\ 0 & 0 & \sin\alpha_{n} & 0 & \cos\alpha_{n} & 0 & 0 & 0 & 0 & 0 & 0 & 0 & 0 & 0\\ 0 & 0 & 0 & 0 & 0 & 0 & 1 & 0 & 0 & 0 & 0 & 0 & 0 & 0\\ 0 & 0 & 0 & 0 & 0 & 0 & 0 & 0 & 1 & 0 & 0 & 0 & 0 & 0\\ 0 & 0 & 0 & 0 & 0 & 0 & 0 & 0 & 0 & 0 & \cos\alpha_{n} & 0 & \sin\alpha_{n} & 0\\ 0 & 0 & 0 & 0 & 0 & 0 & 0 & 0 & 0 & 0 & -\sin\alpha_{n} & 0 & \cos\alpha_{n} & 0 \end{array}\right]. \end{array}$$
